# Dietary macronutrient composition impacts gene regulation in adipose tissue

**DOI:** 10.1038/s42003-024-05876-5

**Published:** 2024-02-16

**Authors:** Kathryn M. Farris, Alistair M. Senior, Débora R. Sobreira, Robert M. Mitchell, Zachary T. Weber, Lars R. Ingerslev, Romain Barrès, Stephen J. Simpson, Angela J. Crean, Marcelo A. Nobrega

**Affiliations:** 1 Department of Human Genetics, University of Chicago, Chicago, IL 60637 USA; 2 Charles Perkins Centre, The University of Sydney, Sydney, NSW 2006 Australia; 3 School of Life and Environmental Sciences, University of Sydney, Sydney, NSW 2006 Australia; 4 Novo Nordisk Foundation Center for Basic Metabolic Research, University of Copenhagen, DK-2200 Copenhagen, Denmark; 5 Institut de Pharmacologie Moléculaire et Cellulaire, Université Côte d’Azur & Centre National pour la Recherche Scientifique (CNRS), Valbonne, 06560 France

**Keywords:** Gene regulation, Fat metabolism

## Abstract

Diet is a key lifestyle component that influences metabolic health through several factors, including total energy intake and macronutrient composition. While the impact of caloric intake on gene expression and physiological phenomena in various tissues is well described, the influence of dietary macronutrient composition on these parameters is less well studied. Here, we use the Nutritional Geometry framework to investigate the role of macronutrient composition on metabolic function and gene regulation in adipose tissue. Using ten isocaloric diets that vary systematically in their proportion of energy from fat, protein, and carbohydrates, we find that gene expression and splicing are highly responsive to macronutrient composition, with distinct sets of genes regulated by different macronutrient interactions. Specifically, the expression of many genes associated with Bardet-Biedl syndrome is responsive to dietary fat content. Splicing and expression changes occur in largely separate gene sets, highlighting distinct mechanisms by which dietary composition influences the transcriptome and emphasizing the importance of considering splicing changes to more fully capture the gene regulation response to environmental changes such as diet. Our study provides insight into the gene regulation plasticity of adipose tissue in response to macronutrient composition, beyond the already well-characterized response to caloric intake.

## Abstract (web summary)

A Nutritional Geometry study in mice identifies complex gene regulatory responses to differences in dietary macronutrient contribution including differential splicing of thousands of genes, most of which did not undergo gene expression changes.

## Introduction

Diet and nutrition are key determinants of metabolism, with implications for both personal and public health. However, what defines a metabolically healthy diet remains elusive. Some studies have focused on total energy and caloric restriction as the most impactful components of a healthy diet^1,2^, while others have argued for the importance of particular nutrients such as fat, carbohydrates, or protein^3,4^. One contested component of a healthy diet is therefore macronutrient composition, namely the ratio of fat, carbohydrates, and protein in a diet. Therefore, understanding the impact of these macronutrients on metabolic health is important for defining a healthy lifestyle and may lead to better-informed nutritional guidelines.

Studies that investigate the impact of diet on metabolic health often rely on a high-fat diet paradigm, where a high-fat, energy-dense diet is compared to a control diet. This study design focuses on a single macronutrient and conflates changes in macronutrient composition with changes in the energy density of the diet. The Nutritional Geometry framework moves beyond this single-macronutrient-at-a-time paradigm by considering a wide range of diets that vary systematically in their ratios of fat, carbohydrates, and protein^5–8^. By considering a large number of isocaloric diets, this framework allows us to determine the metabolic impact of each individual macronutrient and interactions between macronutrients, while controlling for caloric density through titrating indigestible cellulose. Previous work using the Nutritional Geometry framework has shown that both total energy intake and dietary macronutrient composition impact metabolic health, lifespan, and fertility^9–12^, but the mechanisms underlying these effects are not fully known.

A deeper understanding of the molecular mechanisms underlying changes in metabolic function in response to dietary macronutrient composition may provide insights into what constitutes a healthy diet and possible interventions to maintain a healthy metabolic profile. One mechanism that may underlie the observed changes in metabolic function is changes in gene regulation in metabolic tissues, which may lead to changes in tissue function and overall health. Adipose tissue is a key metabolic tissue that is highly functionally dynamic in response to metabolic change^13,14^ and is known to have dynamic gene regulation after exposure to high fat diet^15^. By investigating gene regulation changes in adipose tissue in response to differences in macronutrient composition, we can gain further insights into the impact of diet on adipose tissue function and possibly undercover mechanisms underlying previously reported effects of dietary macronutrients on metabolic health.

Many studies that consider gene regulation change focus on gene expression alone, but it is important to also consider the role of other forms of gene regulation, such as alternative splicing, in the metabolic response to diet. Alternative splicing is a fundamental source of functional complexity in tissues and contributes to tissue identity and development^16,17^. Alternative splicing is also a highly regulated process, and its misregulation can lead to developmental defects and disease^18,19^. However, in the context of gene regulation under environmental effects such as diet, splicing remains relatively understudied compared to other mechanisms of gene regulation, such as transcriptional regulation.

Here, we used the Nutritional Geometry framework to investigate the effects of dietary macronutrient composition on metabolic function and gene regulation in the fat pads of male mice. This framework provides insight into a more complete dietary space than previously considered, allowing us to determine the impact of each macronutrient singly and in combination. Using RNA-seq data collected from the fat pads of mice fed one of ten isocaloric diets *ad libitum*, we identified extensive differences in both gene expression and splicing in response to dietary composition and determined the primary macronutrients driving the observed differences. The majority of alternative splicing events we identified are in genes whose expression is not significantly different in response to dietary composition, highlighting a pervasive and complementary mechanism by which cells regulate their transcriptome beyond regulation of gene expression. Using this comprehensive dietary paradigm we are able to cluster the gene regulation changes on the basis of their functional response to macronutrients and identify several common patterns of gene regulation associated with dietary macronutrient composition, providing insight into the effect of different macronutrients and macronutrient interactions on adipose tissue function.

## Results

### Body composition and metabolic health

**Fig. 1:**
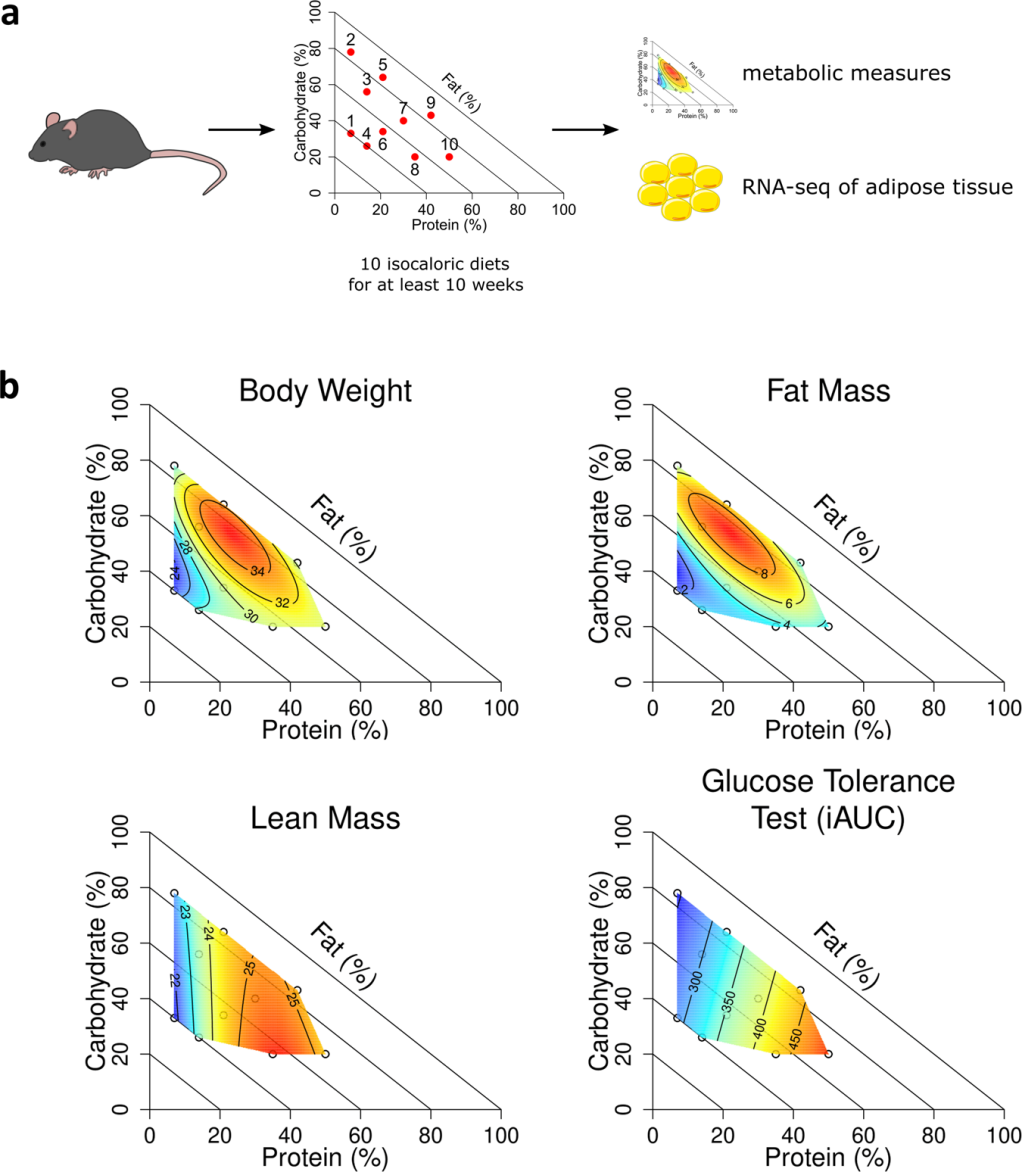
Metabolic response to dietary macronutrient composition. **a** A diagram of the experimental setup and data collection. **b** Surfaces of metabolic measures across the 10 diets plotted as a right-angled mixture triangle, with color indicating the level of the measured variable (red = higher, blue = lower) and isolines showing the model predicted response. The diagonal lines are included to help visualize fat content and are isolines of dietary fat content. At the origin, dietary fat content is 100% and it decreases to 0% as you move away from the origin along the y = x line. *n* = 6 mice per diet. In **a**, the image of a mouse was sourced from SciDraw and was created by Heath Robinson, and is licensed under a CC-BY 4.0 license. Modifications were made. The icon of an adipose cell was sourced from Bioicons and created by Servier, and is licensed under a CC-BY 3.0 Unported License. Modifications were made.

To measure the impact of dietary macronutrient composition on metabolic health, we fed 60 male mice one of 10 isocaloric diets that differed systematically in their ratios of protein, carbohydrates, and fat (Fig. 1a, Table 1). For each mouse, we collected data on body composition, including body weight, fat mass, and lean mass, as well as other measures of metabolic health such as glucose tolerance (Supplementary Fig. 1, Supplementary Data 1). To analyze these data we used a mixture-model framework, where models were fitted for each metabolic response over the dietary space, exploring linear, non-linear, and interactive effects of the macronutrients. Predictions from fitted models were then plotted as a right-angled mixture triangle with the percent dietary protein on the x-axis, percent dietary carbohydrate on the *y*-axis, and percent dietary fat as the distance from the hypotenuse to the origin^20^.

**Table 1:** Dietary macronutrient composition

Diet	Protein (%)	Carbohydrate (%)	Fat (%)
1	7	33	60
2	7	78	15
3	14	56	30
4	14	26	60
5	21	64	15
6	21	34	45
7	30	40	30
8	35	20	45
9	42	43	15
10	50	20	30

The macronutrient composition of each experimental diet as a percent of total energy.

Using this framework, we found that dietary macronutrient composition had a significant impact on body composition and metabolic health (Fig. 1b). Body weight, fat mass, lean mass, and glucose tolerance all differed significantly across the diets. Body weight and fat mass were both maximized on a moderate fat, moderate carb, and moderate protein diet (diet 7, Supplementary Table 1) and minimized on a low protein, moderate carb, high fat diet (diet 1, Supplementary Table 1). When considering associations with single macronutrients, body weight was positively correlated with protein content (*r* = 0.26, *P* = 0.043) and negatively correlated with fat content (*r* = −0.40, *P* = 0.0017) whereas fat mass was positively correlated with carbohydrate content (*r* = 0.40, *P* = 0.0015) and negatively correlated with fat content (*r* = −0.39, *P* = 0.0019) (Supplementary Fig. 2).

In contrast, lean mass was positively correlated with protein content (*r* = 0.52, *P* = 2.0e-05) and not correlated with fat or carbohydrate content (Supplementary Fig. 2). Lean mass was maximized on a moderate protein, high carb, and low fat diet (diet 5) and minimized on low protein diets (diets 1 and 2) (Supplementary Table 1). Glucose tolerance was also impacted by differences in dietary macronutrient composition (Fig. 1b). The incremental area under the curve (iAUC) in an oral glucose tolerance test was positively correlated with protein content (*r* = 0.40, *P* = 0.0017) and negatively correlated with carbohydrate content (*r* = −0.28, *P* = 0.033), but not correlated with fat content (Supplementary Fig. 2).

Dietary macronutrient composition therefore had diverse impacts on body composition and metabolic health. Using the Nutritional Geometry framework, we are able to determine the impact of differences in macronutrient composition alone, in the absence of differences in caloric density. This allows us to ask more precise questions about the impact of individual macronutrients on metabolic health without confounding with the energy density of the diet, as is common in a high fat diet context. In this context, we found evidence for both linear and non-linear effects of dietary macronutrients on various metabolic measures. Body weight and fat mass were both negatively correlated with fat content, with some interactions with protein and carbohydrate content as well. On the other hand, lean mass and glucose tolerance were correlated with protein content but not fat content. Overall, in an isocaloric context the ratio of dietary macronutrients significantly altered the metabolic profiles of these mice.

### Changes in gene regulation in response to diet

**Fig. 2:**
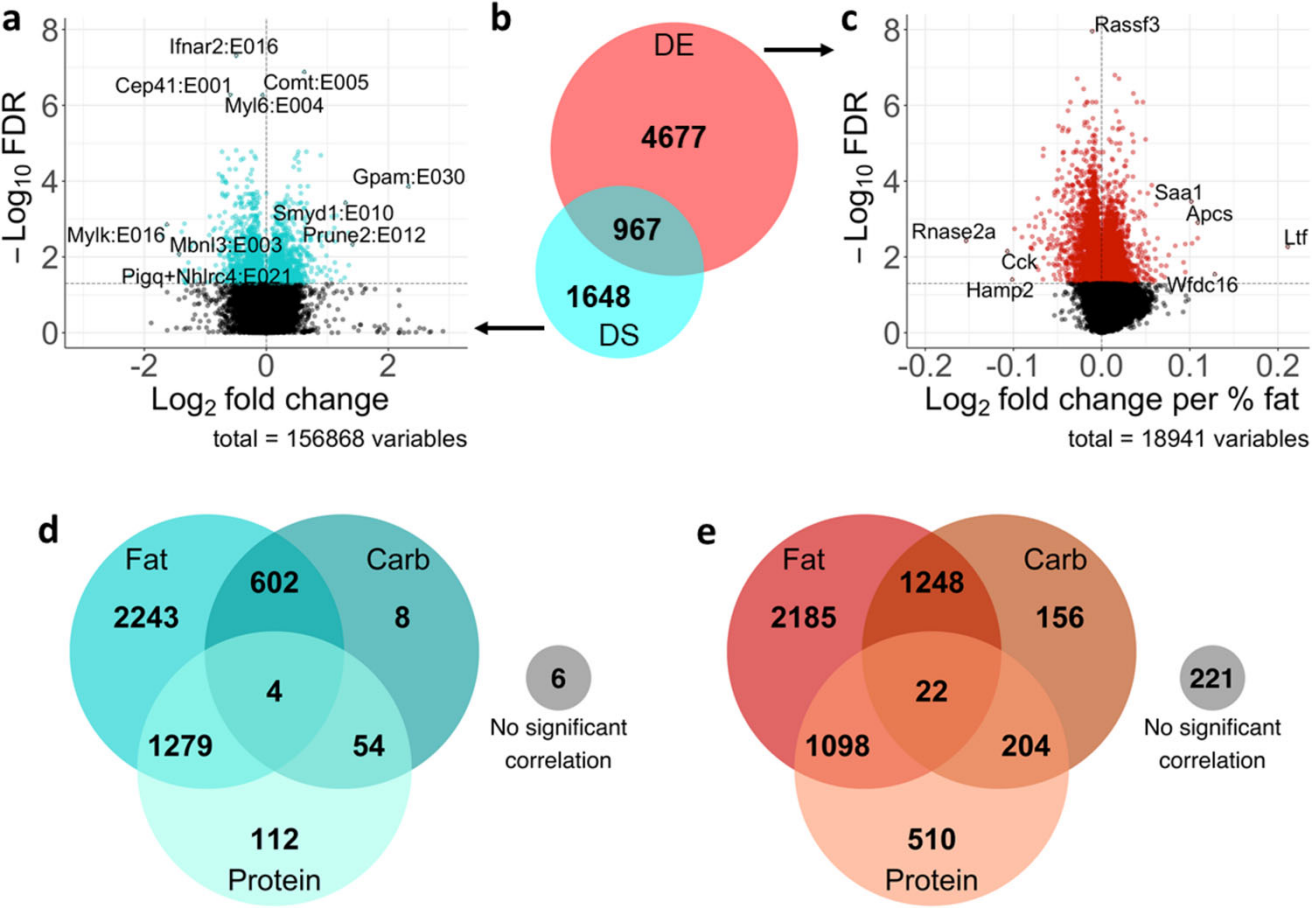
Significant regulatory response to dietary macronutrient composition. **a** Volcano plot of differential splicing changes, plotting the log fold change between 15% dietary fat and 60% dietary fat. Blue dots are significant, black are non-significant. Extreme exons in terms of log fold change or *p*-value are labeled. **b** Venn diagram of differentially expressed and/or differentially spliced genes. **c** Volcano plot of differential expression changes, plotting the log fold change per percent dietary fat. Red dots are significant, black are non-significant. **d** Venn diagram of the correlation of each differentially spliced exon with the three macronutrients. **e** Venn diagram of the correlation of each differentially expressed gene with the three macronutrients. *n* = 57 mice.

To better understand how differences in dietary macronutrient composition led to the observed effects on metabolic health in vivo, we investigated alterations in gene regulation programs associated with the observed changes in body composition and metabolic parameters. We performed RNA-seq in the inguinal fat pads of each of the 60 mice to measures changes in gene expression and splicing across the 10 diets. Following quality assessment, 57 samples were retained for all genomics analyses (*n* = 5 or 6 per diet). We tested the response of each gene or exon across the macronutrient space, and found that there were 4308 differentially spliced exons in 2615 unique genes (Fig. 2a, Supplementary Fig. 3a, Supplementary Data 2) and 5644 differentially expressed genes (Fig. 2c, Supplementary Fig. 3b, Supplementary Data 2). Only 967 genes were both differentially expressed and differentially spliced, with the majority of genes that underwent gene regulatory changes being acted on by only one of the two measured mechanisms (Fig. 2b).

We therefore observed abundant changes in gene regulation in response to differences in dietary macronutrient composition, with expression and splicing changes largely occurring in distinct genes, underscoring how distinct gene regulatory strategies may impact the transcriptome in cells quantitatively (through transcription regulation) and qualitatively (through differential usage of exons encoding specific protein domains). This analysis identifies genes and exons that were significantly impacted by diet, but doesn’t provide insight into what macronutrient or macronutrients these genes and exons might be responding to. We found that metabolic measures can have disparate responses to macronutrient composition (Fig. 1b), and therefore sought to better understand what macronutrient interactions might be driving the observed changes in gene expression and splicing.

### Correlation between gene regulation changes and individual macronutrients

To quantify the impact of individual macronutrients on gene expression and splicing, we calculated the correlation of each differentially expressed gene or differentially spliced exon with fat, protein, and carbohydrate content. We found that dietary fat content is the predominant driver of the observed gene expression and splicing changes (Fig. 2d, e). This was particularly true for the differential splicing changes, where 4128 differentially spliced exons in 2510 genes (96% of all differentially spliced exons) were correlated with dietary fat content (Fig. 2d). Of note, the diets in this study contained varying amounts of non-digestible cellulose to maintain their caloric density. Cellulose content of the diet is positively correlated with fat content, so it is possible that some of the gene regulation changes that are correlated with fat content are actually responding to fiber content of the diet. However, due to the indigestible nature of the cellulose we believe that fat content is the main driver of the observed gene regulation changes.

Although fat content was the strongest driver of gene regulation changes, protein and carbohydrate content were also correlated with many of the changes in gene expression and splicing, often in conjunction with fat content. Gene regulation changes correlated with more than one macronutrient represent 46% of all differentially expressed genes, and 45% of all differentially spliced exons. While correlations with individual macronutrients can capture some of the dynamics of this dietary space, they do not necessarily capture the full response of genes and exons across all 10 diets, especially for gene regulation changes that respond to multiple macronutrients or interactions between macronutrients. To better capture these complex dietary responses, we sought to categorize the gene regulation changes that we observed in terms of their holistic response across the nutrient space encompassed by the 10 diets, as opposed to focusing on each macronutrient separately.

### Clustering analysis of differentially spliced exons

**Fig. 3:**
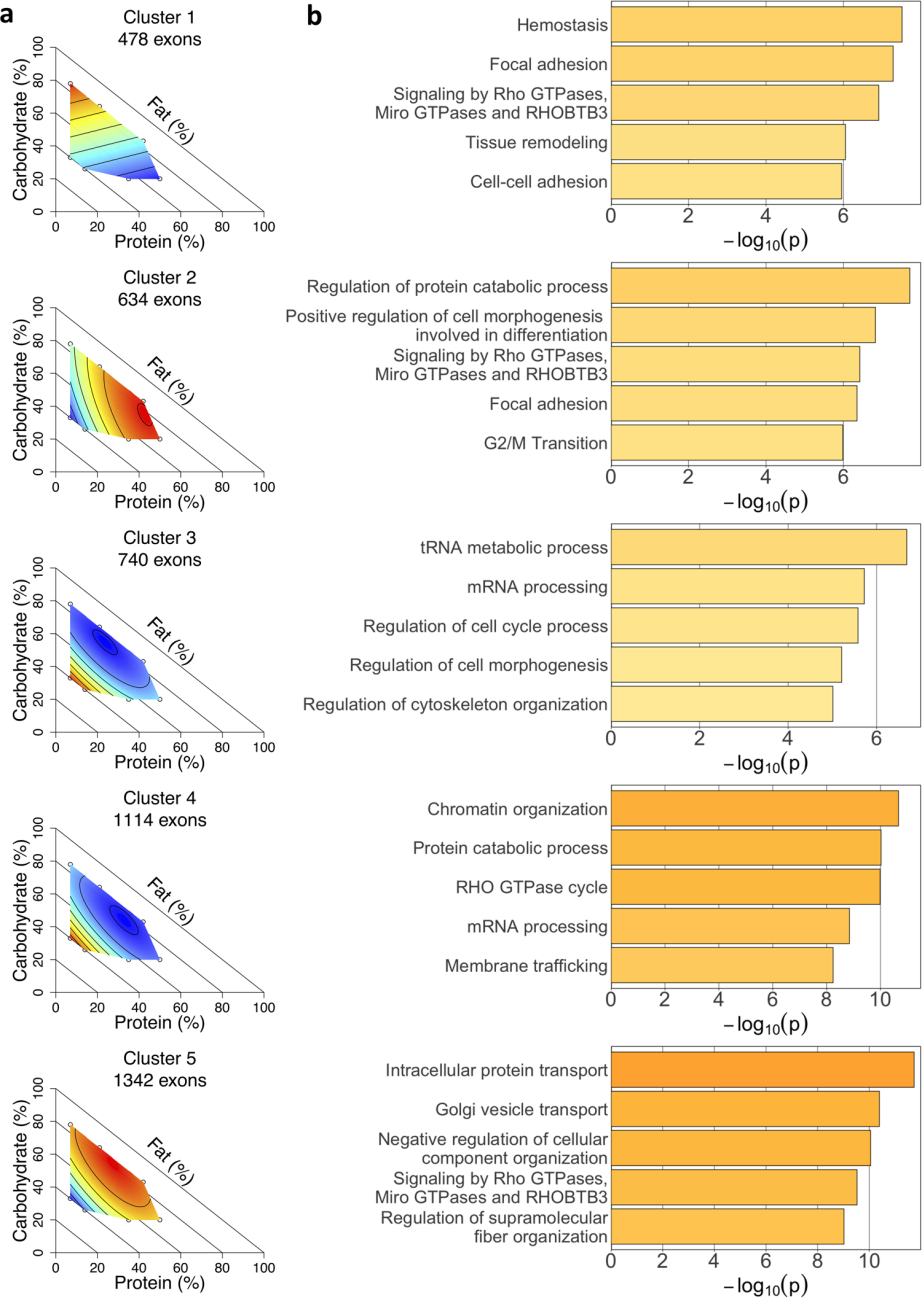
Differential splicing changes clustered into five distinct groups. **a** Surfaces generated from the mean centered and scaled exon usage of each exon assigned to the cluster, with color indicating the level of exon usage (red = higher, blue = lower). **b** The five most significantly enriched functional terms for each cluster. *n* = 6 for diets 1, 5, 6, 7, 8, 9, and 10 and *n* = 5 for diets 2, 3, and 4.

We identified complex gene regulation responses to differences in dietary macronutrient composition, including many genes and exons that responded to multiple macronutrients or possibly interactions between macronutrients. To better partition these complex responses, we can quantify the response of each differentially spliced exon to macronutrient composition using the regression coefficients from a mixture model. Nutritional Geometry then allows these responses to be visualized as response surfaces (topologies) mapped onto dietary macronutrient space. We therefore clustered all the differentially spliced exons based on the regression coefficients for all three nutrients using fuzzy c-means clustering and visualized each cluster using a response surface generated from the mean exon usage for all exons assigned to that cluster. Using this method, we can observe a more representative range of dietary response landscapes than simple linear correlations with individual macronutrients. The results of this analysis with five clusters are shown (Fig. 3a).

Consistent with the results from the correlation analysis, we see that the three largest clusters (clusters 3–5) show a predominant response to fat content, either positive (clusters 3 and 4, which were closely similar in topology) or negative (cluster 5). The remaining two clusters capture interaction effects, namely a positive carbohydrate by negative protein gradient (cluster 1) and a positive protein by negative fat gradient (cluster 2). These interaction effects were not identified as strong signals from the single-nutrient correlation analyses (Fig. 2d) and would most likely have been missed had we not considered the full response of each exon across nutrient space and instead considered one macronutrient at a time, as is conventional.

We next investigated whether these groups of exons that were clustered based on their response to diet also fell into shared functions or pathways. Using functional enrichment analysis^21^, we found that the clusters were significantly enriched for distinct functional terms (Fig. 3b). Cluster 1, which demonstrated a primarily carb by protein gradient, was enriched for terms related to cell adhesion, such as cell-cell adhesion and focal adhesion. The protein by fat cluster (cluster 2) was also enriched for focal adhesion, but showed stronger enrichment for regulation of cell morphogenesis involved in differentiation and regulation of protein catabolic processes. In contrast, the exons responding more to fat content, such as cluster 5, show enrichment for terms related to intracellular transport and organization.

Overall, we observe distinct functional enrichment in groups of exons that respond differently to dietary macronutrient composition, demonstrating the importance of capturing the full dietary response to understand gene regulation changes in response to diet. Further, most of these genes would not have been identified as undergoing gene regulation change if we had considered expression differences alone, emphasizing the need to consider splicing as well as expression changes when analyzing gene regulation responses.

### Differential splicing of key adipocyte genes

In addition to considering the differential splicing changes at the level of clusters and functional groups, we also identified individual splicing events predicted to have significant impact on adipocyte function. These include differential splicing events in *Vegfa* and *Igf1*.

**Fig. 4:**
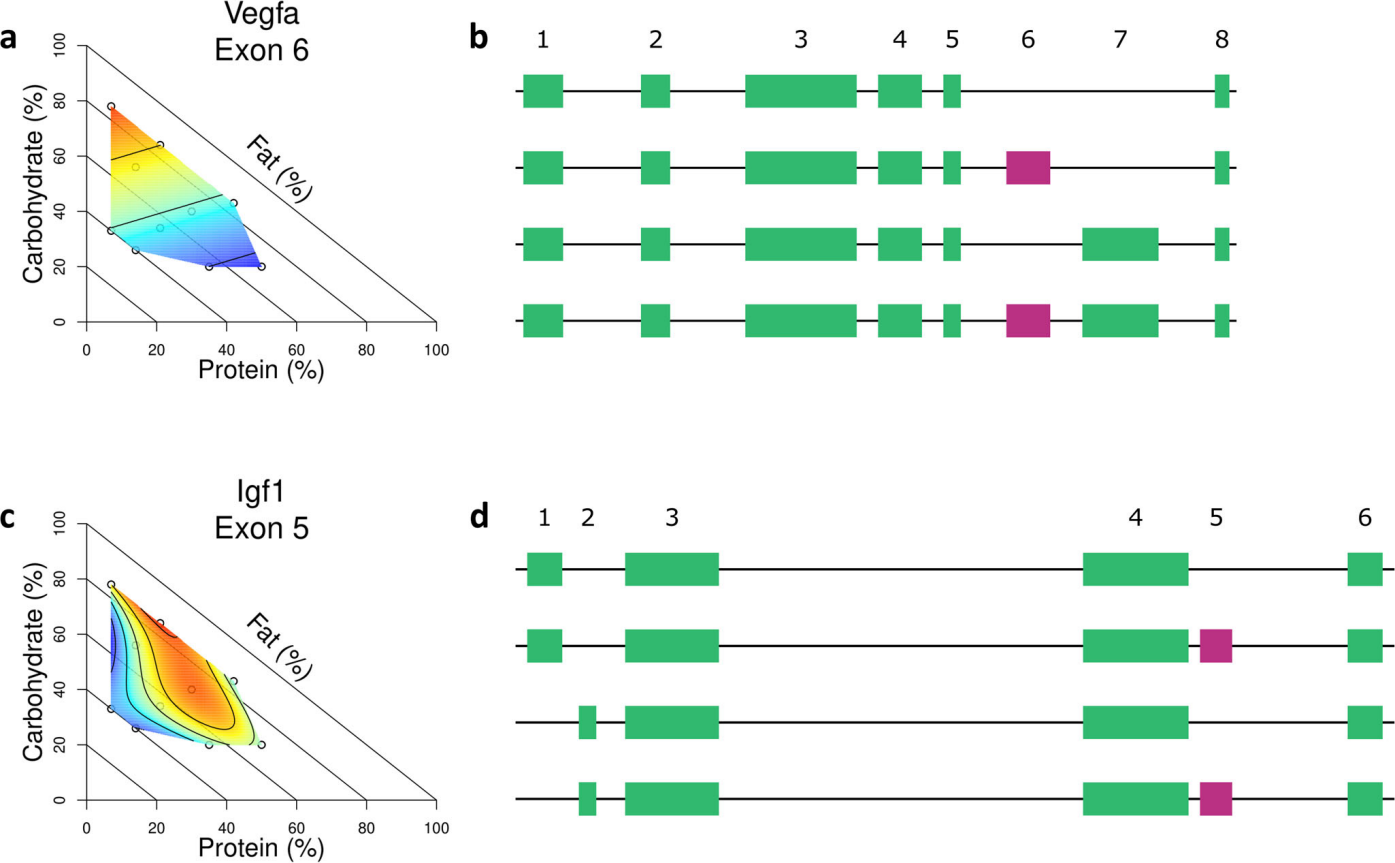
Differential splicing of *Vegfa* and *Igf1*. **a** Surface of the centered and scaled exon usage of *Vegfa* exon 6. **b** Diagram of selected *Vegfa* isoforms. Exon 6 is highlighted in pink. **c** Surface of the centered and scaled exon usage of *Igf1* exon 5. **d** Diagram of selected *Igf1* isoforms. Exon 5 is highlighted in pink. *n* = 6 for diets 1, 5, 6, 7, 8, 9, and 10 and *n* = 5 for diets 2, 3, and 4.

*Vegfa* regulates angiogenesis, and has been implicated in adipose tissue response to diet-induced obesity^22,23^. Specifically, overexpression of *Vegfa* in the fat pads of mice leads to increased vascularization and a healthier phenotype in response to high fat diet^23^. In our study, we found that exon 6 of *Vegfa* was differentially spliced in response to dietary composition, and demonstrated a predominant response to carb and protein content of the diet (Fig. 4a). Exons 6 and 7 contain heparin-binding domains, and are known to be differentially spliced to produce isoforms that contain one, both, or neither domain^24^ (Fig. 4b). Transcripts lacking exon 6 and 7 produce a variant of *Vegfa* that does not bind heparin and is fully soluble, whereas heparin-binding variants of *Vegfa* bind to the cell surface and extracellular matrix, with different isoforms leading to different sites of angiogenesis^25^. Therefore, differential splicing of exon 6 in response to macronutrient composition suggests that changes in dietary carb and/or protein content may lead to changes in the angiogenic potential and patterns of fat tissue.

Another key adipocyte gene that we identified as differentially spliced is *Igf1. Igf1* regulates adipocyte differentiation^26,27^ and controls the response of adipose tissue to metabolic stress^28^. Alternative splicing produces isoforms of IGF1 that differ in their N-terminus (known as the signal peptide) and C-terminus (known as the E peptide), which are removed post-transcriptionally to produce the same mature peptide^29^. In mice, there are two main variants of the E peptide based on whether exon 5 is spliced in or out^29^ (Fig. 4d). The E peptide that includes exon 5 has been functionally implicated in IGF1 bioavailability, *via* more strongly facilitating IGF1 binding to the extracellular matrix than the shorter E peptide^30^, and increases the adipogenic potential of bone marrow mesenchymal stem cells^31^. Here, we found that exon 5 of *Igf1* was differentially spliced in response to dietary composition, and identified a specific response to the protein by fat ratio of the diet (Fig. 4c).

Although adipose tissue does express *Igf1*, it is not the main source of circulating IGF1. Rather, the main contributor to circulating IGF1 levels is hepatocytes^32^. To further support our finding that dietary macronutrient composition significantly impacts *Igf-1* splicing and adipose tissue function, we therefore asked whether *Igf-1* splicing in the liver was also responsive to diet. Using liver samples collected from the same 60 mice in which we assayed adipose gene regulation changes, we used RNA-seq to measure the impact of dietary macronutrient composition on *Igf-1* splicing in the liver. We found that *Igf-1* exon 5 is indeed differentially spliced in response to diet in the liver, and responds primarily to protein content of the diet (Supplementary Fig. 4). The *Igf-1* exon 5 splicing surfaces are distinct between adipose and liver tissue, with the liver splicing displaying a more marked response to dietary protein content. Notably, in both tissues exon 5 of *Igf-1* is responsive to diet and minimized on low protein diets. This result suggests that there may be differences in IGF1 bioavailability in response to different dietary macronutrient compositions in both liver and adipose tissue, potentially leading to changes in adipose tissue function.

Since neither *Vegfa* nor *Igf1* were differentially expressed in response to dietary macronutrient composition, the impact of macronutrient composition on these biological processes would not have been detected by measuring differential expression. By analyzing differential splicing changes, we were able to detect gene regulation changes that may alter adipocyte function and that would have been missed when considering gene expression alone.

### Clustering analysis of differentially expressed genes

**Fig. 5:**
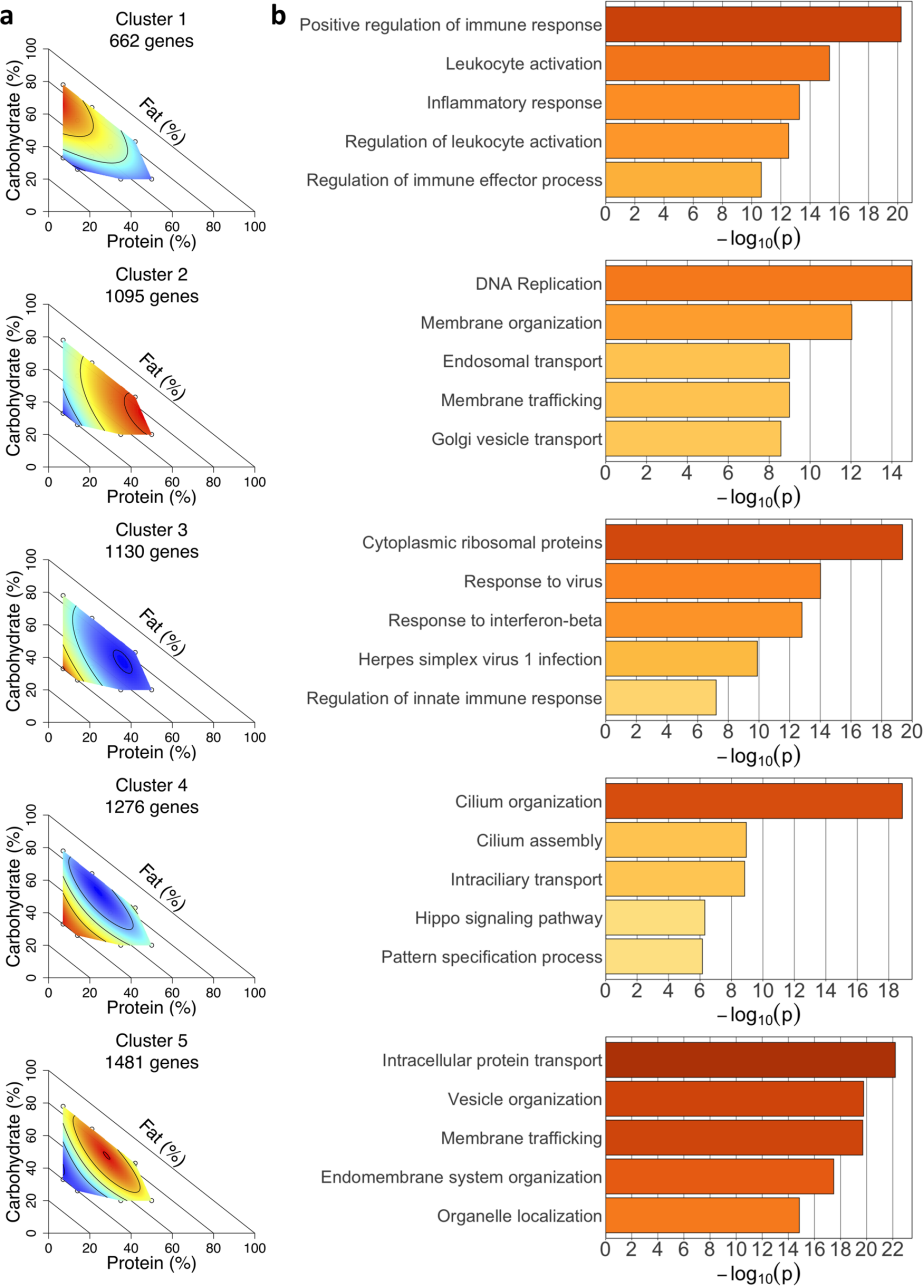
Differential expression changes clustered into five distinct groups. **a** Surfaces generated from the mean centered and scaled expression of each gene assigned to the cluster, with color indicating the level of expression (red = higher, blue = lower). **b** The five most significantly enriched functional terms for each cluster. *n* = 6 for diets 1, 5, 6, 7, 8, 9, and 10 and *n* = 5 for diets 2, 3, and 4.

Next, we performed fuzzy c-means clustering on the responses of the 5644 differentially expressed genes to capture the differential expression dynamics across all ten diets. The results of this analysis with five clusters are shown (Fig. 5a). From these five clusters, the largest two clusters show a strong positive (cluster 4) or negative (cluster 5) fat gradient. This is consistent with the single-nutrient correlation results that identified fat content as the strongest driver of the observed expression changes (Fig. 2e), as well as the splicing clustering results in which the three largest clusters (clusters 3–5) also show a predominant response to fat (Fig. 3a). The remaining three clusters capture interaction effects between the macronutrients, specifically fat by protein gradients (cluster 2 showing a negative fat by positive protein interaction and cluster 3 showing a positive fat by negative protein interaction) and a positive carbohydrate by negative protein gradient (cluster 1). Again, these dietary interactions were not apparent in single nutrient analyses.

We next investigated whether genes falling into these distinct clusters based on their response to diet also had distinct biological functions. Using functional enrichment analysis, we found that the clusters differed not just in their response to diet but also in their functional enrichment (Fig. 5b). One particularly strong enrichment signal was for immune function in genes assigned to cluster 1, possibly representing an inflammatory phenotype associated with carb and protein content in the diet. We found another strong enrichment signal for cilium function in cluster 4, where the enriched categories included cilium organization, cilium assembly, and intraciliary transport. Differentiating preadipocytes are ciliated, and cilia function is known to be involved in adipocyte differentiation^33,34^. The observed gene expression differences in cilia function could therefore indicate changes in adipogenic potential in the fat pad in response to dietary fat content. Overall, these data demonstrate that there are distinct sets of genes that respond differently to dietary macronutrient composition and carry out distinct functions in adipose tissue.

### Changes in the expression of cilium-associated genes in response to dietary fat

One of the most striking enrichment signals that arose from the clustering analysis of differentially expressed genes was the enrichment in cluster 4 for genes involved in ciliary function (Fig. 5b). When we investigated this signal more closely, we found that the signal was driven in part by a set of genes associated with Bardet-Biedl syndrome (BBS). BBS is an autosomal recessive ciliopathy with symptoms that include obesity^35^. At least 19 genes have been shown to cause BBS, many of which are associated with a structure called the BBSome, which is a protein complex that is involved in protein trafficking to the cilium^36^.

**Fig. 6:**
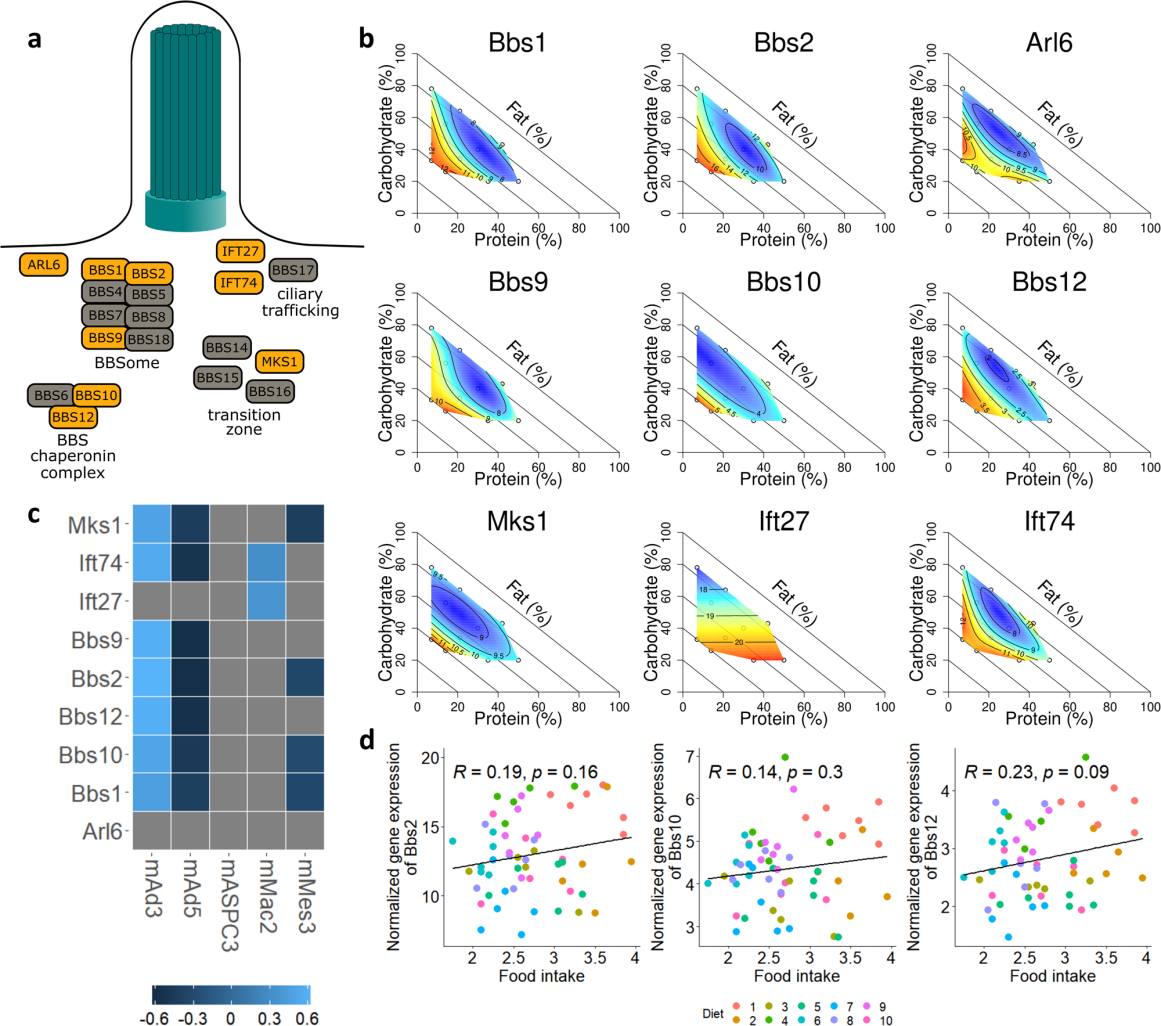
Response of BBS genes to dietary fat. **a** Diagram of the primary cilia, with BBS genes organized by their role in cilia function. Differentially expressed BBS genes are highlighted in orange. BBS-associated gene functions adapted from previous work^35,64,65^. **b** Surfaces generated from the expression of each differentially expressed BBS gene. **c** Heatmap of the correlation of each differentially expressed BBS gene’s expression with the cell type proportions estimated by deconvolution. **d** Plots of the correlation between food intake (grams per day) and the expression of *Bbs2*, *Bbs10*, and *Bbs12*. *n* = 6 for diets 1, 5, 6, 7, 8, 9, and 10 and *n* = 5 for diets 2, 3, and 4.

In our differential expression analysis, we found that nine BBS-associated genes were differentially expressed in response to dietary macronutrient composition. These nine genes have a variety of ciliary-related functions, including some that are components of the BBSome itself (Fig. 6a). Eight of the nine differentially expressed BBS-associated genes were assigned to cluster 4 and one (*Ift27*) was assigned to cluster 3 (Fig. 5a). As expected from the clustering analysis, the surface plots for each individual gene assigned to cluster 4 demonstrated a strong expression response to dietary fat content (Fig. 6b). This may represent a novel association of BBS genes with diet-induced metabolic changes, in particular in response to differences in dietary fat content.

Many BBS genes are known to be upregulated during adipocyte differentiation^37^. It is therefore possible that the increase of BBS gene expression in response to dietary composition represents a change in cell type composition of the fat pads, with more differentiating preadipocytes present in diets with high fat content. We therefore sought to determine if there were changes in cell type composition associated with changes in BBS gene expression. All analyses in this study were done in bulk tissue samples, and we do not have direct access to measurements of cell type composition of the tissue. Using the dampened weighted lease squares (DWLS) method^38^, we performed cellular deconvolution to computationally estimate the cell type composition of each bulk tissue sample based on bulk gene expression and a reference single-cell RNA-seq dataset of mouse adipose tissue^39^. We found that there were significant differences in predicted cell type composition in response to dietary composition, including a change in the estimated proportion of adipocyte progenitor cells that was predominately associated with protein content in the diet (Supplementary Fig. 5).

We next asked whether BBS gene expression was correlated with cell type composition. We limited this analysis to the five cell types that were identified at >1% frequency in at least one sample. We found that there were correlations between BBS gene expression and predicted cell type composition (Fig. 6c). In particular, seven of the nine differentially expressed BBS genes were positively correlated with mAd3 proportion and negatively correlated with mAd5 proportion. Although this correlation may suggest an association between BBS gene expression and these specific adipose subclusters, it may also simply be due to the fact that both BBS genes and the proportion of adipocyte subclusters are independently correlated with fat content in the diet. These mouse adipocyte subclusters have previously been identified as responding to high fat diet, with mAd3 proportion reduced after high fat diet and mAd5 increased^39^. Here, we saw a negative association between mAd5 proportion and fat content, and a positive association between mAd3 proportion and fat content (Supplementary Fig. 5). Given that our diets were isocaloric, our results suggest that these adipocyte subclusters may be responding to caloric density rather than fat content in the high fat diet context. Notably, none of the differentially expressed BBS genes were associated with the predicted proportion of adipocyte progenitors, suggesting that the observed gene expression changes are not due to a change in cell type composition.

To complement and extend these results based on computational deconvolution of cell type composition, we also performed single-nucleus RNA-seq in adipose tissue from one mouse from a diet with high fat content (diet 4, 60% fat) and one mouse from a diet with low fat content (diet 7, 30% fat). After anchoring the resulting single-nucleus datasets to the same single-cell atlas that was used for deconvolution^39^, we identified clusters associated with all of the major cell types we expected to find in adipose tissue, such as adipocytes, adipose stem and progenitor cells (ASPCs), immune cells, and mesothelial cells (Supplementary Fig. 6a). We do observe some differences in cell type composition between these two samples, most notably a decrease in the proportion of total cells identified as adipocytes in the diet 7 sample as compared to diet 4.

To assess the expression of the differentially expressed BBS genes in each sample and cluster, we calculated a BBS expression score based on the sum of the normalized, centered, and scaled expression across the nine BBS-associated genes of interest. As expected from the bulk expression results, we see that the BBS genes are overall more lowly expressed in the diet 7 sample than in diet 4 (Supplementary Fig. 6b), with a particular depletion of BBS gene expression in the adipocyte and mesothelial cell clusters in diet 7. Overall, these results suggest that BBS gene expression is lowered in multiple cell types, including adipocytes and ASPCs, in response to differences in dietary fat content, possibly leading to altered adipogenic potential in response to diet.

Finally, some BBS genes, such as *Bbs2*^40^, *Bbs10*^41^, and *Bbs12*^42^, are associated with changes in food intake. We therefore tested the association between the expression of these BBS genes and the food intake of each individual mouse. We found that there were no significant correlations (Fig. 6d), suggesting that in this context BBS gene expression is not a significant driver of food intake.

Altogether, our results indicated that the observed changes in BBS gene expression may regulate ciliary function in response to dietary macronutrient composition, which does not appear to be caused by changes in cell type composition in adipose tissue. This suggests a possibly novel role for the BBSome and other BBS-associated genes in response to diet.

## Discussion

In this study, we aimed to use the Nutritional Geometry framework to dissect the effects of dietary composition and interactions between macronutrients on gene regulation in adipocytes. By using ten isocaloric diets that cover a large range of the macronutrient space, we could precisely assign the observed gene regulation changes to specific macronutrient gradients and control for effects of caloric density and energy intake. Using this framework, we have generated a comprehensive analysis of gene regulation changes in fat tissue in response to differences in macronutrient composition, and identified key changes that may be important for adipocyte biology.

Our results illustrate the power of the Nutritional Geometry framework to identify patterns of regulation beyond linear relationships with single macronutrients. Using RNA-seq data from a broad range of diets, we are able to quantify the holistic response of each gene regulation change to macronutrient composition and cluster the gene regulation responses across nutrient space. This clustering analysis identified a positive carbohydrate by negative protein gradient (cluster 1) and a positive protein by negative fat gradient (cluster 2) as key patterns in both the differential splicing and differential expression analyses (Figs. 3a, 5a). Interaction effects such as these would be difficult or impossible to identify from single nutrient analyses or a standard high fat diet paradigm. Clustering gene regulation responses across the nutrient space encompassed by these 10 diets therefore allows for more precise interpretation of response to diet than was previously possible.

We also showed that both expression and splicing in fat tissue are dynamic in response to environmental change such as dietary composition, and that they act on largely separate gene sets (Fig. 2). Many studies of gene regulatory responses focus solely on gene expression changes, and in doing so may miss a great deal of impactful regulatory changes that are due to alternative splicing. Here, we see large changes in the transcriptome in response to macronutrient composition that are regulated at the level of alternative splicing and would not have been detected by looking at gene expression alone. Of note, it is possible that the lack of overlap between gene expression and splicing changes is due to lower power to detect changes at the splicing level than at the expression level. However, if that were the case we would expect the splicing results to largely be a subset of the expression results, whereas we identify more genes that are acted on solely by splicing than genes that are impacted by both splicing and expression. We therefore conclude that these findings suggest that thousands of differentially spliced exons represent a concerted cellular response to dietary composition that impacts the transcriptome in a mechanism independent of gene expression, highlighting the importance of giving further consideration to the role of alternative splicing in adipocyte biology and in the response of other tissues to dietary composition.

Our results also have implications for the interpretation of the effects of genetic variants on metabolic traits and diseases. Metabolic disorders such as obesity and diabetes are complex diseases, with both genetic and environmental components^43,44^. When considering the contribution of genetics to these diseases, previous studies have demonstrated that splicing quantitative trait loci (sQTLs) play an important role in disease risk and etiology^45–47^. While some genetic variants have been associated with gene expression and metabolic traits^48–51^, the functional impact of most GWAS variants associated with metabolic disease remains uncharacterized^52–54^. We found that splicing is highly dynamic in response to dietary composition, including alternative splicing changes that may have significant impacts on adipocyte biology. One possible mechanism underlying these uncharacterized GWAS variants is therefore that they modulate splicing, but not gene expression, in response to environmental inputs such as diet. These results highlight the role of splicing in the response of adipose tissue to dietary composition, and provide a foundation from which to consider the impact that genetic variation may have on these processes.

Further research will be required to determine the similarity between the changes seen here and the gene regulatory impact of differences in macronutrient composition in humans. Of particular interest is determining whether there may be any genotype by environment interactions governing these responses in humans, with implications for public dietary guidelines and personalized medicine approaches. Of note, this study was conducted in only young male mice of a single strain, and future studies are needed to determine whether these results replicate fully in both female mice and humans.

Finally, although the diets used in this study were isocaloric per gram of food (i.e. all of the diets had the same energy density), the mice were *ad libitum* fed and there was variation in food intake (and therefore energy intake) across the diets, as show in Fig. 1d in Crean et al.^55^. In particular, mice on the lowest protein diets (7% of energy from protein) showed increased food intake, as previously reported^9,56^. Although we cannot rule out the possibility that some of the gene regulation changes we see are due to differences in energy intake between the diets, food intake was not significantly correlated with fat content (Supplementary Fig. 7), which we identified as the strongest driver of gene regulation changes in this study. As this study focused on isocaloric diets, further research is necessary to consider the impact of energy level on these processes.

Overall, this study utilizes the Nutritional Geometry framework to expand our understanding of the impact of macronutrient composition on metabolic function and gene regulation in adipose tissue. We find that both expression and splicing are highly dynamic across the dietary space, and that considering multiple modes of gene regulation change provides novel insights into the processes underlying the metabolic response to macronutrient composition.

## Methods

### Animal husbandry

C57BL/6 J male mice (*n* = 60), housed in the Charles Perkins Centre (Sydney, Australia) animal facility (24-26◦C, 44-46% humidity, 12 h day/light cycle), were used in this study. Four-week-old mice were purchased from the Animal Resources Centre (Murdoch, Australia) and allowed to acclimate for 3 days before being randomly assigned to dietary treatments. Food and water were supplied ad libitum, mice were weighed weekly, and health checks performed at least twice weekly. Mice were anaesthetized with sodium pentobarbital (100 mg/kg) and culled at 21 weeks of age for tissue collection. Gonadal white adipose tissue deposits and liver tissue deposits were weighed, snap frozen in liquid nitrogen, and stored at -80◦C until further use. All procedures were reviewed and approved by the University of Sydney animal ethics committee (project number 2019/1610). We have complied with all relevant ethical regulations for animal use.

### Diets

Ten treatment diets, manufactured by Specialty Feeds (Glen Forrest, Australia), were designed to include ingredients of AIN-93G in varying proportions to cover the full range of physiologically viable macronutrient intake space (Fig. 1). Non-digestible cellulose was included at varying amounts to maintain the net metabolizable energy of diets at 14.7 MJ/kg (3.5 kcal/g). Micronutrient content was equal across diets. Protein content was exome-matched to the *Mus musculus* genome^57^, achieved by mixing casein and whey protein isolates supplemented with leucine, threonine, methionine, tyrosine, phenylalanine, tryptophan, alanine, aspartic acid, arginine, glycine, histidine and serine. Omega 3 to omega 6 fatty acid ratio was maintained at 1:3.7 using a combination of soybean oil, linseed oil and lard, with saturated fats making up 23.2% of dietary fats. Carbohydrate sources included wheat starch, dextrinised starch and sucrose at a ratio of 4: 1.3: 1. Individual food intake was measured at 16 and 20 weeks of age by weighing food before and after a 24- h feeding period. Bedding was changed at the start of intake measures and sifted for food crumbs at the end of the feeding period to obtain as accurate measures of food consumed as possible.

### Body composition and metabolic phenotyping

Metabolic phenotyping was completed at 18 weeks of age. Body composition was measured using an EchoMRI-900-A130 (EchoMRI, Houston, USA). Oral glucose tolerance tests were performed after 4 h of fasting. Blood samples were obtained by tail tipping and blood glucose measured using a clinical glucometer (Accu-Chek Performa, Roche Diagnostics Australia Pty Ltd). Glucose (2 g/kg lean mass) was administered via oral gavage and blood glucose was measured at baseline, 15, 30, 45, 60 and 90 min. Blood from tail tipping was also used to measure blood insulin at baseline, 15 and 30 min, using an enzyme-linked immunosorbent assay (ELISA) following manufacturer’s instructions (Crystal Chem IL).

### Bulk RNA extraction and sequencing

Fat tissue was lysed with a 20 G needle in Trizol (Life Technologies, #15596018) and total RNA was extracted using the Zymo Direct-zol RNA Miniprep kit (Zymo, #R2052). RNA quantity and quality were measured using the Agilent 2100 Bioanalyzer (Agilent). RNA-seq libraries were generated from 1 μg of total RNA using the NEBNext Ultra II Directional RNA library prep kit (NEBNext, #E7765) and NEBNext Poly(A) mRNA magnetic isolation module (NEBNext, #E7490) with a size selection step to generate 300 bp inserts. The libraries were sequenced using an Illumina NovaSeq 6000 machine (Illumina) with 100 bp paired end reads. Samples were sequenced to an average depth of 84,769,220 reads per sample (54,667,206–209,155,949).

10 - 20 mg of flash-frozen liver tissue was disrupted using a Dounce homogenizer in ice-cold homogenization buffer (250 mM sucrose, 25 mM KCl, 5 mM MgCl2, 20 mM Tricine pH 7.8). The homogenate was mixed 1:3 with Tri-Reagent (Zymo) and passed through a 20g-syringe 10X. The Zymo Direct-zol RNA Microprep kit (Zymo) was used to isolate total RNA according to the manufacturer’s recommendations. RNA integrity and concentration was assessed with the Agilent Bioanalyzer Nano kit (Agilent) and 1 ug of mRNA was reverse transcribed and amplified using the NEB Ultra II Directional RNA library prep kit (NEBNext). Sequencing was performed on an Illumina NovaSeq 6000 (Illumina) with 50 bp paired end reads. Samples were sequenced to an average depth of 25,421,980 reads per sample (11,447,079 – 54,008,386).

### Response surfaces

Response surfaces were created by fitting a series of mixture models (a.k.a, Scheffe’s polynomials) to each response variable in R using the mixexp package. We started by fitting a null model (i.e., intercept only; y ~ 1), before also fitting a linear and a non-linear mixture model, equivalent to equations 1 and 2 in Lawson and Willden^58^. We then selected among models using Akaike Information Criterion, where the simplest model (i.e., fewest terms) within 2 points of the minimal AIC score was selected. In the event that a non-null model was favored we infer an effect of the diet composition on the outcome of interest. To visualize the effects to diet composition we created response surfaces by taking the predicted values from AIC-favored models, and projecting them in to the right-angle mixture triangle (RMT) compositional space^20^. AIC values and a summary of the selected model are provided for all variables in Supplementary Data 3.

### Differential expression and splicing analysis

RNA-seq reads were aligned to the GRCm39 genome using STAR two-pass mapping^59^ and read counts per gene were quantified. For the adipose tissue samples, on average 93.67% of reads per sample uniquely mapped to the genome, with a range of 89.39%–94.53%, resulting in an average of 79,365,634 mapped reads per sample (51,015,437–196,899,410). For the liver tissue samples, on average 83.82% of reads per sample uniquely mapped to the genome, with a range of 78.91%–90.20%, resulting in an average of 21,322,851 mapped reads per sample (9,577,376–43,882,813). Sample quality was assessed using principal components analysis (PCA) and three samples were removed from all genomics analyses in adipose tissue. To test for differential expression, we used edgeR^60^ and treated the percent fat and percent carbohydrates in the diets as continuous variables, testing the model~*percent fat+percent carbohydrates*. As the percent fat, carbohydrates, and proteins in each diet always sum to 100 the third macronutrient is redundant. Genes with an FDR < 0.05 in this analysis were considered significantly differentially expressed. To test for differential splicing, we used DEXSeq^61^ and exons with an FDR < 0.05 in this analysis were considered significantly differentially spliced. In the liver analysis, RNA collection date was included as a covariate. When considering the overlap between differential expression and splicing, genes with at least one significantly differentially spliced exon were considered to be differentially spliced.

### Macronutrient correlation

To identify genes or exons with a significant correlation with individual macronutrients, we calculated the Pearson’s correlation between the expression of each differentially expressed gene or the exon usage of each differentially spliced exon and the percentage of each macronutrient in the diets. Multiple test correction was performed and correlations with FDR < 0.05 were considered significant.

### Fuzzy c-means clustering

Two separate clustering analyses were performed, one for differentially expressed genes and one for differentially spliced exons. All genes that were significantly differentially expressed or exons that were significantly differentially spliced were included. Genes and exons were clustered based on their model coefficients from fitting the model $\sim 0+{percent\; fat}+{percent\; carbohydrates}+{percent\; protein}$ in edgeR. Model coefficients were centered and scaled to account for differences in expression across genes. Using the e1071 package in R, fuzzy c-means clustering was performed on the centered and scaled model coefficients for every differentially expressed gene or every differentially spliced exon. Genes or exons were assigned to the cluster for which they had the highest membership. Functional enrichment analysis of the gene sets associated with each cluster was performed using Metascape (http://metascape.org, *21*).

### Cell type deconvolution of bulk tissue samples

To estimate the cell type proportions of each bulk tissue sample, we performed cellular deconvolution based on gene expression signatures using the DWLS method^38^ and a single-cell atlas of mouse white adipose tissue^39^. Any cell type that was estimated at > 1% proportion in at least one sample was considered in further analyses. To determine the relationship between BBS gene expression and cell type proportion, Pearson’s correlation with two-sided hypothesis testing was calculated between each differentially expressed BBS gene and each estimated cell type. Multiple test correction was performed via FDR estimation.

### Single nucleus extraction and sequencing

Nuclei were isolated from 200–300 mg of flash-frozen mouse white adipose tissue according to Van Hauwaert, E. L. et al.^62^. Briefly, adipose tissue was finely minced on a petri dish in nuclei isolation buffer (NIB, 250 mM sucrose, 10 mM HEPES, 1.5 mM MgCl2, 10 mM KCl, 0.001% triton x100, 0.2 mM DTT, 0.5U ul RNase inhibitor). Minced tissue was disrupted with a Dounce homogenizer in NIB, filtered through a 70 um cell strainer and nuclei isolated from contaminating lipids and cellular debris by differential centrifugation. Nuclei were suspended in nuclei resuspension buffer (NRB, 1 x PBS with 1% BSA, 2 mM MgCl2, and 0.04 U/ul RNase inhibitor) for 10X genomics. All steps were performed rapidly on ice and all reagents were ice-cold. The resulting suspensions were processed using the Chromium Next GEM Single Cell 3’ Kit v3.1 according to the manufacturer’s instructions (10X Genomics). Barcoding was performed using the Chromium Controller (10X Genomics) and sequencing was performed on an Illumina NovaSeq X (Illumina) with 100-bp paired end reads.

FASTQ files derived from sequencing were aligned to GRCm38/mm10 genome using 10X Cell Ranger v7.1.0. (10X Genomics). Filtered counts matrices were inspected and then re-filtered for minimum total read count of 1500 Unique Molecular Identifiers (UMIs) per nucleus. After filtering, 18,101 cells from the sample from diet 4 and 11,727 cells from the sample from diet 7 were retained for analysis. Count matrices for both sample data sets and for reference nuclei from Emont et al.^39^ were normalized and regressed for cell cycle (s.score, g2m.score) and mitochondrial read percentage using the SCTransform algorithm included as a part of the Seurat R Package^63^. Reference nuclei were subset to include only murine nuclei derived from perigonadal adipose tissue of male mice fed chow diets. Cell-type labels for the two diet samples were learned using the reference dataset using Seurat’s FindTransferAnchors and MapQuery functions. UMAP projections were generated using the first 30 principal components of gene expression computed on the normalized reference dataset.

For each sample data set, BBSome gene expression scores were calculated as the sum of normalized, centered, and scaled expression across nine BBS-associated genes of interest (*Bbs1*, *Bbs2*, *Arl6*, *Bbs9*, *Bbs10*, *Bbs12*, *Mks1*, *Ift27*, and *Ift74*) and plotted by the learned cell type groups for each diet.

### Statistics and reproducibility

Biological replicates were employed in this study, using *n* = 6 per diet for all metabolic measures and the liver genomic analyses and *n* = 5 or 6 per diet for the bulk adipose genomic analyses (*n* = 6 for diets 1, 5, 6, 7, 8, 9, and 10 and *n* = 5 for diets 2, 3, and 4). Statistical analyses were performed using R (v. 4.1.1), specifically the DEXSeq (v 1.40.0), edgeR (v 3.36.0), e1071 (v 1.7–9), mixexp (v 1.2.7), and seurat (v 5.0.0) packages. In the differential expression analysis, lowly expressed genes were filtered out (<10 reads in 53 or more samples) and in the differential splicing analysis, lowly expressed exons were filtered out (<10 reads across all samples). All data exclusions were performed before performing any analyses. Correlations were measured using Pearson’s correlation. Results with an FDR < 0.05 were considered significant.

### Reporting summary

Further information on research design is available in the Nature Portfolio Reporting Summary linked to this article.

### Supplementary information

Supplementary Material

Description of Additional Supplementary Files

Supplementary Data 1

Supplementary Data 2

Supplementary Data 3

Reporting Summary

## Data Availability

All RNA-seq data generated by this study is available through SRA (bulk and snRNA-seq adipose samples: PRJNA987348, liver samples: PRJNA1043119). All other source data have been provided in the Supplementary Information and Supplementary Data.
