# Supplementary material for: Dietary macronutrient composition impacts gene regulation in adipose tissue
Source: Commun Biol. 2024 Feb 16;7:194. doi: 10.1038/s42003-024-05876-5 (PMC10873408; doi:10.1038/s42003-024-05876-5)
Supplement: Supplementary file 1 — Supplementary Material [file 42003_2024_5876_MOESM1_ESM.docx]

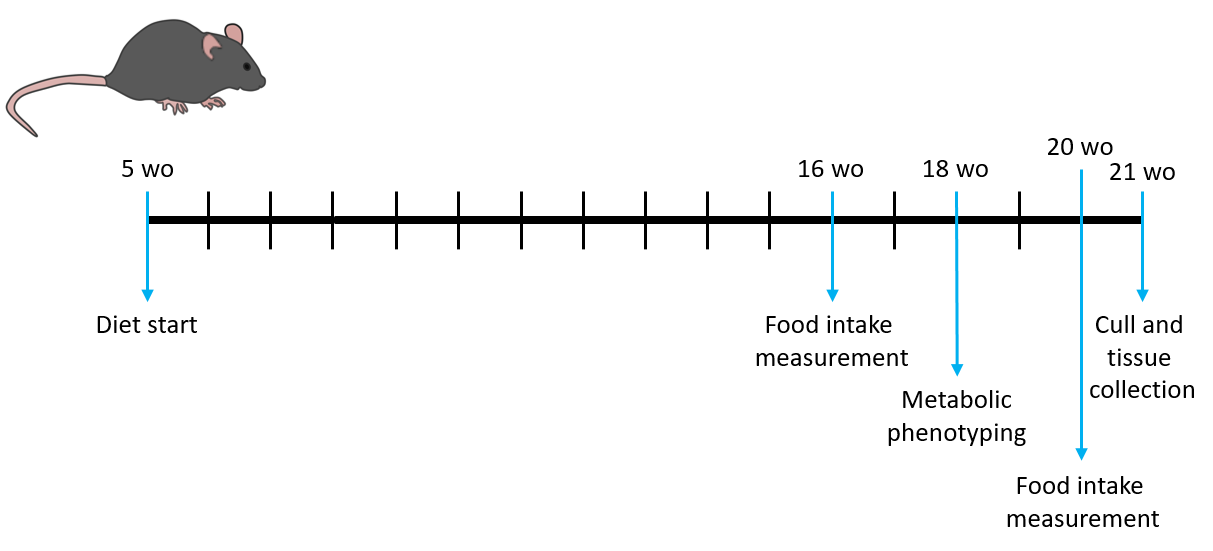


Supplementary Figure 1. **Experimental timeline.** Timeline showing the age of the mice (in weeks) when each measurement or intervention was carried out (wo = weeks old). The image of a mouse was sourced from SciDraw and was created by Heath Robinson, and is licensed under a CC-BY 4.0 license. Modifications were made.


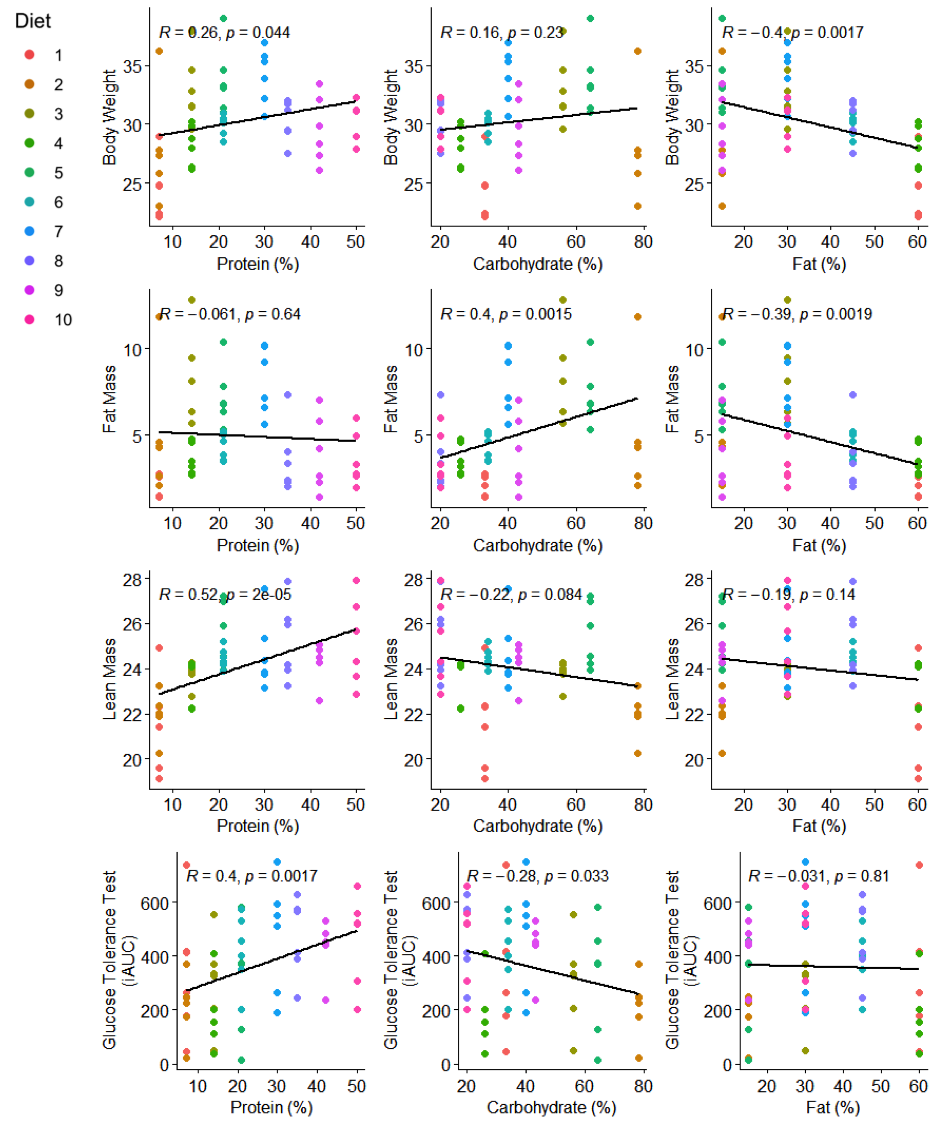


Supplementary Figure 2. **Correlations of metabolic traits with each dietary macronutrient.** Plots of the correlation of each dietary macronutrient with each metabolic trait in Figure 1. Correlation was assessed using Pearson’s correlation with two-sided hypothesis testing. Each point is a mouse, colored by diet. n = 6 mice per diet.


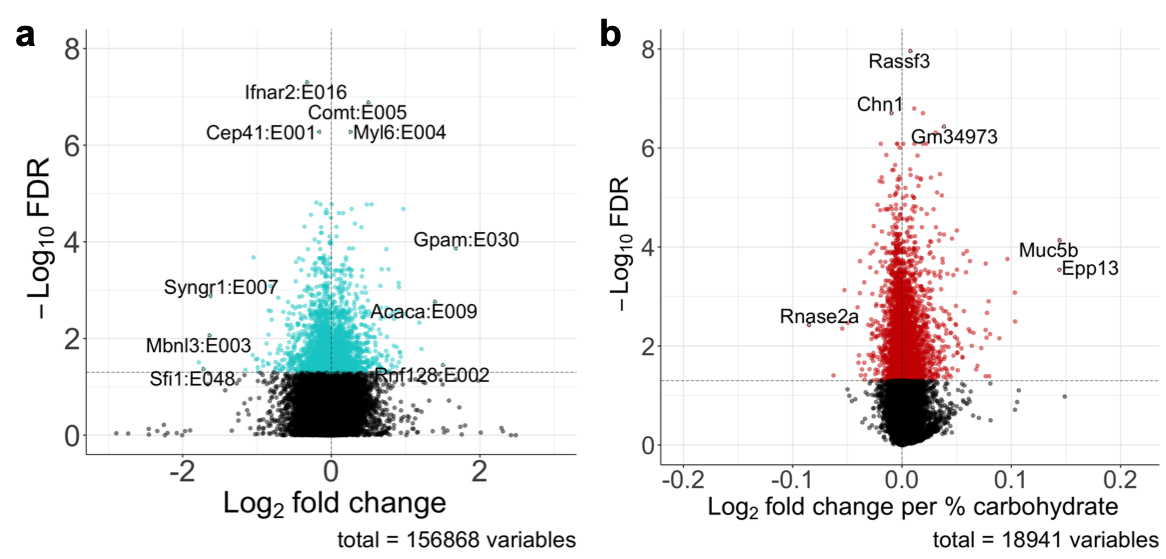


Supplementary Figure 3. **Gene regulatory changes relative to dietary carbohydrates.** A. Volcano plot of differential splicing changes, plotting the log fold change between 20% dietary carbohydrates and 78% dietary carbohydrates. Blue dots are significant, black are non-significant. Extreme exons in terms of log fold change or p-value are labeled. B. Volcano plot of differential expression changes, plotting the log fold change per percent dietary carbohydrates. Red dots are significant, black are non-significant. n = 57 mice.


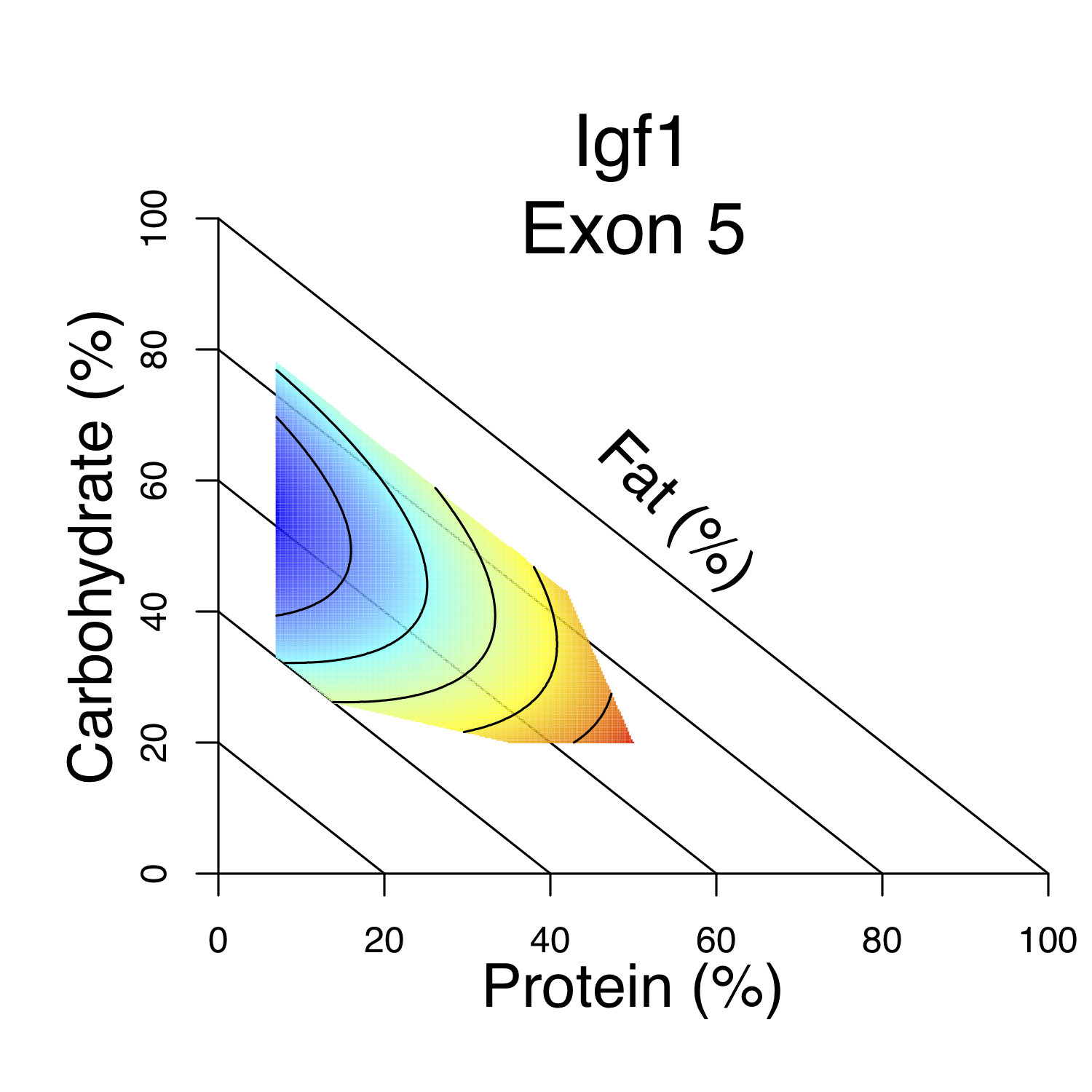


Supplementary Figure 4. **Differential splicing of *Igf1* in the liver.** Surface of the centered and scaled exon usage of *Igf1* exon 5 in the liver. n = 6 mice per diet.


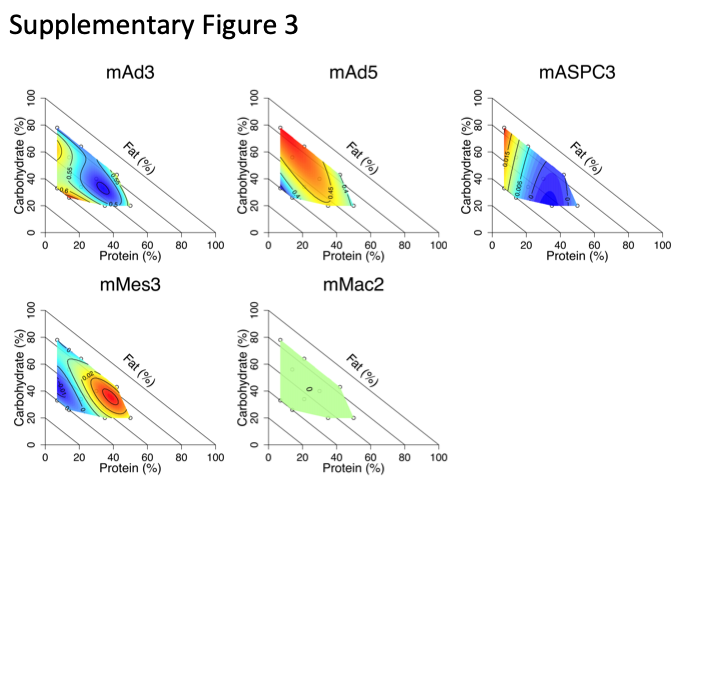


Supplementary Figure 5. **Computationally estimated cell type proportions.** Surfaces generated from the computationally estimated cell type proportions of each fat tissue sample. For the proportion of macrophages (mMac2), the data supported a null model with no impact of dietary macronutrients. n = 6 for diets 1, 5, 6, 7, 8, 9, and 10 and n = 5 for diets 2, 3, and 4


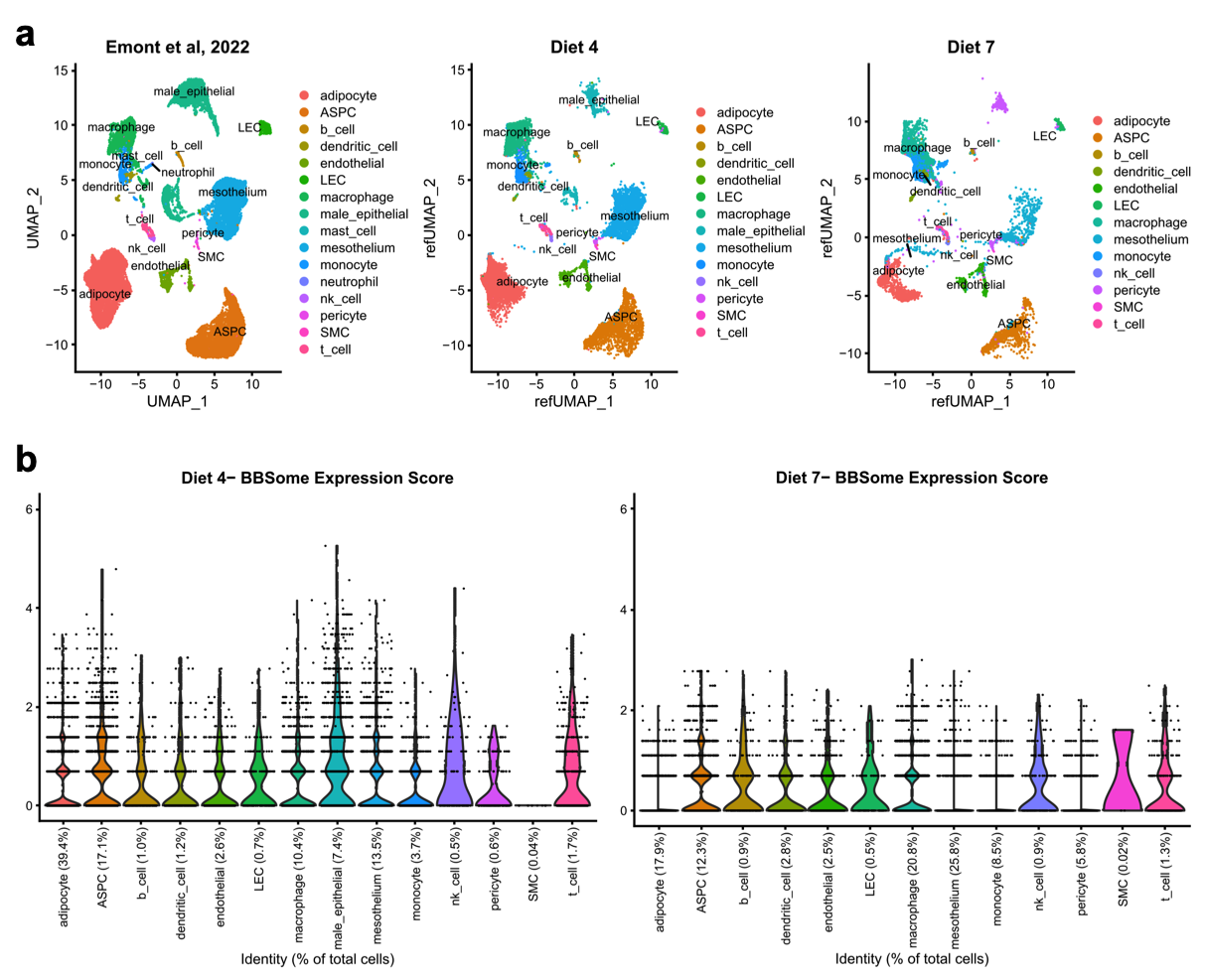


Supplementary Figure 6. **Single nucleus RNA-seq results from diets 4 and 7.** A. UMAP projections of the reference dataset (perigonadal adipose tissue from chow-fed male mice from Emont et al. (*39*)), the diet 4 cells, and the diet 7 cells. Each point is a cell, colored by cell type. B. Violin plots of the BBSome expression score for each cell type in diet 4 and diet 7. The expression score is based on the nine BBS genes that were identified as differentially expressed in the bulk analysis.


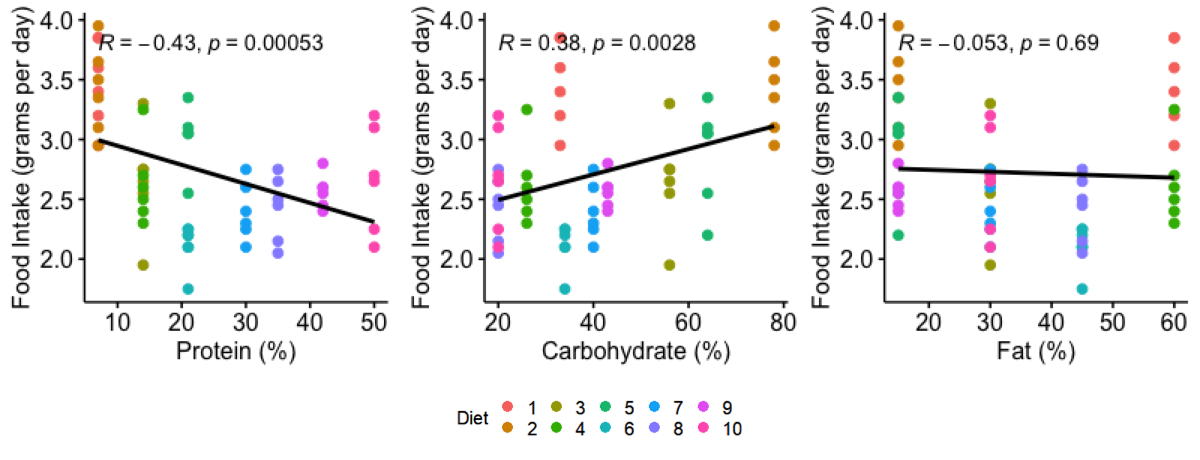


Supplementary Figure 7. **Correlations of food intake with each dietary macronutrient.** Plots of the correlation of each dietary macronutrient with food intake. Correlation was assessed using Pearson’s correlation with two-sided hypothesis testing. Each point is a mouse, colored by diet. n = 6 mice per diet.

Supplementary Table 1. **Metabolic trait values.** Mean and standard deviation for the measurements of each metabolic trait in Figure 1 in each diet. n = 6 mice per diet.

| Diet | Body Weight | Fat Mass | Lean Mass | Glucose iAUC |
| --- | --- | --- | --- | --- |
| 1 | 24.7 ± 2.5 | 2.04 ± 0.55 | 21.6 ± 2.1 | 343 ± 240 |
| 2 | 28.0 ± 4.4 | 4.95 ± 3.52 | 21.9 ± 1.0 | 214 ± 115 |
| 3 | 33.0 ± 2.9 | 8.03 ± 2.80 | 23.8 ± 0.5 | 307 ± 170 |
| 4 | 28.2 ± 1.7 | 3.58 ± 0.89 | 23.5 ± 1.0 | 186 ± 125 |
| 5 | 33.7 ± 2.9 | 7.23 ± 1.74 | 25.5 ± 1.4 | 320 ± 211 |
| 6 | 29.9 ± 0.9 | 4.28 ± 0.74 | 24.5 ± 0.4 | 420 ± 133 |
| 7 | 34.1 ± 2.3 | 8.15 ± 1.95 | 24.7 ± 1.6 | 476 ± 211 |
| 8 | 30.2 ± 1.7 | 3.57 ± 2.01 | 25.2 ± 1.8 | 469 ± 145 |
| 9 | 29.5 ± 2.8 | 3.88 ± 2.20 | 24.3 ± 0.9 | 397 ± 127 |
| 10 | 30.4 ± 1.6 | 3.59 ± 1.53 | 25.2 ± 1.9 | 462 ± 171 |

Supplementary Data 1. **Metabolic and food intake measures.**

Supplementary Data 2. **Differential splicing and expression results for all genes in adipose tissue.**

Supplementary Data 3**. AIC values and mixture model summaries for all variables.**
