# Supplementary material for: Dietary macronutrient composition impacts gene regulation in adipose tissue
Source: Commun Biol. 2024 Feb 16;7:194. doi: 10.1038/s42003-024-05876-5 (PMC10873408; doi:10.1038/s42003-024-05876-5)
Supplement: Supplementary file 2 — Description of Additional Supplementary Files [file 42003_2024_5876_MOESM2_ESM.pdf]

Description of Additional Supplementary Files

File name: Supplementary Data 1
Description: Metabolic and food intake measures.

File name: Supplementary Data 2
Description: Differential splicing and expression results for all genes in adipose tissue.

File name: Supplementary Data 3
Description: AIC values and mixture model summaries for all variables.
