# Supplementary material for: Dietary macronutrient composition impacts gene regulation in adipose tissue
Source: Commun Biol. 2024 Feb 16;7:194. doi: 10.1038/s42003-024-05876-5 (PMC10873408; doi:10.1038/s42003-024-05876-5)
Supplement: Supplementary file 6 — Reporting Summary [file 42003_2024_5876_MOESM6_ESM.pdf]

nature portfolio

Corresponding author(s): Marcelo Nobrega, Romain Barrès, Angela Crean, Kathryn Farris

Last updated by author(s): Dec 19, 2023

Reporting Summary

Nature Portfolio wishes to improve the reproducibility of the work that we publish. This form provides structure for consistency and transparency in reporting. For further information on Nature Portfolio policies, see our Editorial Policies and the Editorial Policy Checklist.

Statistics

For all statistical analyses, confirm that the following items are present in the figure legend, table legend, main text, or Methods section.

n/a	Confirmed	
☐	☒	The exact sample size (n) for each experimental group/condition, given as a discrete number and unit of measurement
☐	☒	A statement on whether measurements were taken from distinct samples or whether the same sample was measured repeatedly
☐	☒	The statistical test(s) used AND whether they are one- or two-sided<br>*Only common tests should be described solely by name; describe more complex techniques in the Methods section.*
☐	☒	A description of all covariates tested
☐	☒	A description of any assumptions or corrections, such as tests of normality and adjustment for multiple comparisons
☐	☒	A full description of the statistical parameters including central tendency (e.g. means) or other basic estimates (e.g. regression coefficient) AND variation (e.g. standard deviation) or associated estimates of uncertainty (e.g. confidence intervals)
☐	☒	For null hypothesis testing, the test statistic (e.g. F, t, r) with confidence intervals, effect sizes, degrees of freedom and P value noted<br>*Give P values as exact values whenever suitable.*
☒	☐	For Bayesian analysis, information on the choice of priors and Markov chain Monte Carlo settings
☒	☐	For hierarchical and complex designs, identification of the appropriate level for tests and full reporting of outcomes
☐	☒	Estimates of effect sizes (e.g. Cohen's d, Pearson's r), indicating how they were calculated

Our web collection on statistics for biologists contains articles on many of the points above.

Software and code

Policy information about availability of computer code

Data collection	No software used for data collection
Data analysis	STAR (v 2.6.1a), Metascape (v 3.5), Cell Ranger (v. 7.1.0), R (v 4.1.1), R packages: DEXSeq (v 1.40.0), edgeR (v 3.36.0), e1071 (v 1.7-9), mixexp (v 1.2.7), seurat (v 5.0.0)

For manuscripts utilizing custom algorithms or software that are central to the research but not yet described in published literature, software must be made available to editors and reviewers. We strongly encourage code deposition in a community repository (e.g. GitHub). See the Nature Portfolio guidelines for submitting code & software for further information.

Data

Policy information about availability of data

All manuscripts must include a data availability statement. This statement should provide the following information, where applicable:

- Accession codes, unique identifiers, or web links for publicly available datasets
- A description of any restrictions on data availability
- For clinical datasets or third party data, please ensure that the statement adheres to our policy

All RNA-seq data generated by this study is available through SRA (bulk and snRNA-seq adipose samples: PRJNA987348, liver samples: PRJNA1043119). All other raw data has been provided in the supplementary files.

Research involving human participants, their data, or biological material

Policy information about studies with human participants or human data. See also policy information about sex, gender (identity/presentation), and sexual orientation and race, ethnicity and racism.

Reporting on sex and gender	N/A
Reporting on race, ethnicity, or other socially relevant groupings	N/A
Population characteristics	N/A
Recruitment	N/A
Ethics oversight	N/A

Note that full information on the approval of the study protocol must also be provided in the manuscript.

Field-specific reporting

Please select the one below that is the best fit for your research. If you are not sure, read the appropriate sections before making your selection.

☒ Life sciences ☐ Behavioural & social sciences ☐ Ecological, evolutionary & environmental sciences

For a reference copy of the document with all sections, see nature.com/documents/nr-reporting-summary-flat.pdf

Life sciences study design

All studies must disclose on these points even when the disclosure is negative.

Sample size	This study was part of a larger study investigating paternal effects and sample size was selected based on previous studies to have sufficient power to detect paternal effects - as described in Crean, A. J. et al, Nat Commun (2023). This sample size is also comparable to previous nutritional geometry studies that measured direct effects, including proteomic differences between diet groups (n = 8, Couteur et al, Cell Metabolism (2021)).
Data exclusions	In the bulk adipose genomic analyses, 3 samples were excluded based on quality-control considerations. For the differential expression analysis, lowly expressed genes were filtered out (less than 10 reads in 53 or more samples). For the differential splicing analysis, lowly expressed exons were filtered out (less than 10 reads across all samples). All data exclusions were performed before performing any analyses.
Replication	Biological replicates were employed in this study, using n = 6 mice per diet for all metabolic measures and liver genomic analyses and n = 5 or 6 mice per diet for adipose genomic analyses (n = 6 for diets 1, 5, 6, 7, 8, 9, and 10 and n = 5 for diets 2, 3, and 4). There were no concerns that findings were not reproducible.
Randomization	Following an acclimation period of 3 days, mice were randomly assigned to a dietary treatment.
Blinding	Investigators were not blinded to group allocation during data collection as the measures used were not subjective.

Reporting for specific materials, systems and methods

We require information from authors about some types of materials, experimental systems and methods used in many studies. Here, indicate whether each material, system or method listed is relevant to your study. If you are not sure if a list item applies to your research, read the appropriate section before selecting a response.

Materials & experimental systems

n/a	Involved in the study	
☒	☐	Antibodies
☒	☐	Eukaryotic cell lines
☒	☐	Palaeontology and archaeology
☐	☒	Animals and other organisms
☒	☐	Clinical data
☒	☐	Dual use research of concern
☒	☐	Plants

Methods

n/a	Involved in the study	
☒	☐	ChIP-seq
☒	☐	Flow cytometry
☒	☐	MRI-based neuroimaging

Animals and other research organisms

Policy information about studies involving animals; ARRIVE guidelines recommended for reporting animal research, and Sex and Gender in Research

Laboratory animals	Four-week-old male C57BL/6J (JAX strain code 000664) mice
Wild animals	No wild animals were used
Reporting on sex	All mice used in this study were male mice
Field-collected samples	No field-collected samples were used
Ethics oversight	All procedures were reviewed and approved by the University of Sydney animal ethics committee (project number 2019/1610)

Note that full information on the approval of the study protocol must also be provided in the manuscript.
